# Ethyl 3-[(3,5-dimethyl­phen­yl)amino­carbon­yl]propanoate

**DOI:** 10.1107/S1600536809029511

**Published:** 2009-07-29

**Authors:** B. Thimme Gowda, Sabine Foro, B. S. Saraswathi, Hartmut Fuess

**Affiliations:** a Department of Chemistry, Mangalore University, Mangalagangotri 574 199, Mangalore, India; b Institute of Materials Science, Darmstadt University of Technology, Petersenstrasse 23, D-64287 Darmstadt, Germany

## Abstract

The non-H atoms in the title compound, C_14_H_19_NO_3_, lie on a mirror plane. The amide O and ester carbonyl O atoms are *trans* to each other. Furthermore, the C=O and O—CH_2_ bonds of the ester group are *syn* with respect to each other. In the crystal, mol­ecules are packed into centrosymmetric dimers through inter­molecular N—H⋯O hydrogen bonds.

## Related literature

For related structures, see: Gowda *et al.* (2009**a* ▶,*b* ▶,c* ▶).
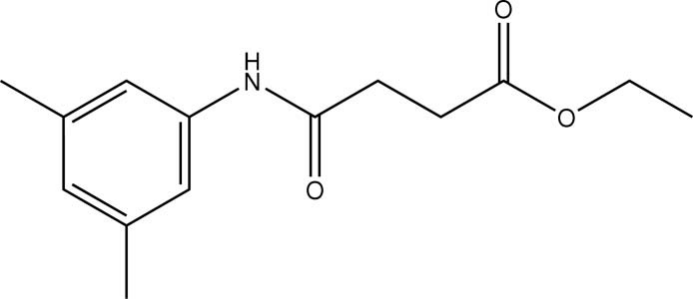


## Experimental

### 

#### Crystal data

C_14_H_19_NO_3_
*M* *_r_* = 249.30
Tetragonal, 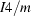

*a* = 19.938 (2) Å
*c* = 7.0367 (9) Å
*V* = 2797.3 (5) Å^3^
*Z* = 8
Cu *K*α radiation
μ = 0.67 mm^−1^
*T* = 299 K
0.40 × 0.28 × 0.25 mm

#### Data collection

Enraf–Nonius CAD-4 diffractometer
Absorption correction: none
4040 measured reflections
1367 independent reflections
1201 reflections with *I* > 2σ(*I*)
*R*_int_ = 0.044
3 standard reflections frequency: 120 min intensity decay: 1.0%

#### Refinement

*R*[*F* ^2^ > 2σ(*F* ^2^)] = 0.058
*wR*(*F* ^2^) = 0.169
*S* = 1.11
1367 reflections
120 parameters
H atoms treated by a mixture of independent and constrained refinement
Δρ_max_ = 0.32 e Å^−3^
Δρ_min_ = −0.32 e Å^−3^

### 

Data collection: *CAD-4-PC* (Enraf–Nonius, 1996 ▶); cell refinement: *CAD-4-PC*; data reduction: *REDU4* (Stoe & Cie, 1987 ▶); program(s) used to solve structure: *SHELXS97* (Sheldrick, 2008 ▶); program(s) used to refine structure: *SHELXL97* (Sheldrick, 2008 ▶); molecular graphics: *PLATON* (Spek, 2009 ▶); software used to prepare material for publication: *SHELXL97*.

## Supplementary Material

Crystal structure: contains datablocks I, global. DOI: 10.1107/S1600536809029511/tk2515sup1.cif

Structure factors: contains datablocks I. DOI: 10.1107/S1600536809029511/tk2515Isup2.hkl

Additional supplementary materials:  crystallographic information; 3D view; checkCIF report

## Figures and Tables

**Table 1:** Hydrogen-bond geometry (Å, °)

*D*—H⋯*A*	*D*—H	H⋯*A*	*D*⋯*A*	*D*—H⋯*A*
N1—H1*N*⋯O2^i^	0.85 (3)	2.15 (3)	2.995 (3)	176 (3)

Symmetry code: (i) 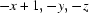.

**Fig. 1.**
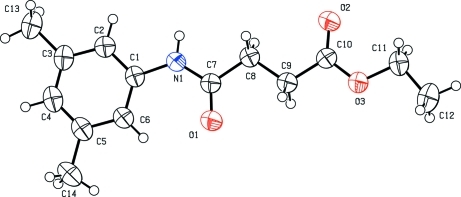
Molecular structure of (I), showing the atom labelling and the displacement ellipsoids are at the 50% probability level. The H atoms are represented as small spheres of arbitrary radii.

**Fig. 2.**
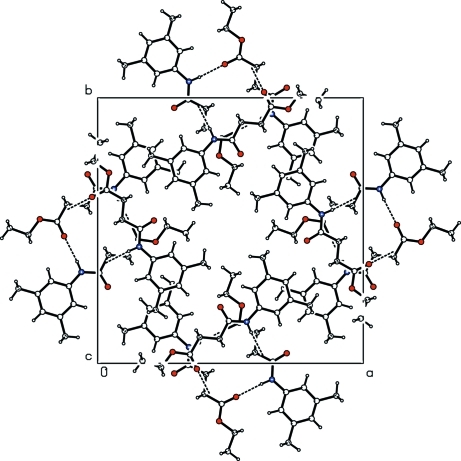
Molecular packing of (I) with hydrogen bonds shown as dashed lines.

## supplementary crystallographic information

### Comment

As a part of studying the effect of ring and side chain substitutions on the structures of the substituted amides (Gowda *et al.*, 2009*a,b,c*), the crystal structure of ethyl *N*-(3,5-dimethylphenyl)succinamate (I) has been determined. The non-hydrogen atoms lie on a crystallographic mirror plane. The conformations of N—H and C=O bonds in the amide segment of the structure are *trans* to each other (Fig. 1). Likewise, the amide-O atom and ester carbonyl-O atoms are *trans* to each other. The C=O and O—CH_2_ bonds of the ester group are in *syn* positions to each other, similar to that observed between the C=O and O—H bonds in the crystal structures of *N*-(2,6-dimethylphenyl)succinamic acid (Gowda *et al.*, 2009*b*) and *N*-(2-chlorophenyl)succinamic acid (Gowda *et al.*, 2009*a*).

The presence of N—H···O hydrogen bonds connect the molecules into centrosymmetric dimers (Table 1).

### Experimental

A solution of succinic anhydride (0.025 mole) in toluene (25 ml) was treated dropwise with a solution of 3,5-dimethylaniline (0.025 mole) also in toluene (20 ml) with constant stirring. The resulting mixture was stirred for about one hour and set aside for an additional hour at room temperature for the completion of reaction. The mixture was then treated with dilute hydrochloric acid to remove the unreacted 3,5-dimethylaniline. The resultant solid *N*-(3,5-dimethylphenyl)succinamic acid was filtered under suction and washed thoroughly with water to remove the unreacted succinic anhydride and succinic acid. *N*-(3,5-Dimethylphenyl)succinamic acid was recrystallized into ethyl *N*-(3,5-dimethylphenyl)succinamate (I) from hot ethanol. The rod like colourless single crystals of (I) were grown in hot ethanolic solution by slow evaporation.

### Refinement

The H atoms of the NH group, of C11 and C12 were located in a difference map and their position refined [N—H = 0.85 (3) Å, C—H = 0.98 (4)–1.03 (3) Å]. The other H atoms were positioned with idealized geometry using a riding model [C—H = 0.93–0.97 Å]. All H atoms were refined with isotropic displacement parameters (set to 1.2 times of the *U*_eq_ of the parent atom).

### Crystal data

**Table:**

C_14_H_19_NO_3_	*D*_x_ = 1.184 Mg m^−^^3^
*M**_r_* = 249.30	Cu *K*α radiation, λ = 1.54180 Å
Tetragonal, *I*4/*m*	Cell parameters from 25 reflections
Hall symbol: -I 4	θ = 4.4–20.5°
*a* = 19.938 (2) Å	µ = 0.67 mm^−^^1^
*c* = 7.0367 (9) Å	*T* = 299 K
*V* = 2797.3 (5) Å^3^	Rod, colourless
*Z* = 8	0.40 × 0.28 × 0.25 mm
*F*(000) = 1072	

### Data collection

**Table:**

Enraf–Nonius CAD-4 diffractometer	*R*_int_ = 0.044
Radiation source: fine-focus sealed tube	θ_max_ = 67.0°, θ_min_ = 3.1°
graphite	*h* = −16→23
ω/2θ scans	*k* = −16→23
4040 measured reflections	*l* = −3→8
1367 independent reflections	3 standard reflections every 120 min
1201 reflections with *I* > 2σ(*I*)	intensity decay: 1.0%

### Refinement

**Table:**

Refinement on *F*^2^	Secondary atom site location: difference Fourier map
Least-squares matrix: full	Hydrogen site location: inferred from neighbouring sites
*R*[*F*^2^ > 2σ(*F*^2^)] = 0.058	H atoms treated by a mixture of independent and constrained refinement
*wR*(*F*^2^) = 0.169	*w* = 1/[σ^2^(*F*_o_^2^) + (0.1022*P*)^2^ + 1.0554*P*] where *P* = (*F*_o_^2^ + 2*F*_c_^2^)/3
*S* = 1.11	(Δ/σ)_max_ = 0.001
1367 reflections	Δρ_max_ = 0.32 e Å^−^^3^
120 parameters	Δρ_min_ = −0.32 e Å^−^^3^
0 restraints	Extinction correction: *SHELXL97* (Sheldrick, 2008), Fc^*^=kFc[1+0.001xFc^2^λ^3^/sin(2θ)]^-1/4^
Primary atom site location: structure-invariant direct methods	Extinction coefficient: 0.0012 (3)

### Special details

**Table:**

Geometry. All e.s.d.'s (except the e.s.d. in the dihedral angle between two l.s. planes) are estimated using the full covariance matrix. The cell e.s.d.'s are taken into account individually in the estimation of e.s.d.'s in distances, angles and torsion angles; correlations between e.s.d.'s in cell parameters are only used when they are defined by crystal symmetry. An approximate (isotropic) treatment of cell e.s.d.'s is used for estimating e.s.d.'s involving l.s. planes.
Refinement. Refinement of *F*^2^ against ALL reflections. The weighted *R*-factor *wR* and goodness of fit *S* are based on *F*^2^, conventional *R*-factors *R* are based on *F*, with *F* set to zero for negative *F*^2^. The threshold expression of *F*^2^ > σ(*F*^2^) is used only for calculating *R*-factors(gt) *etc*. and is not relevant to the choice of reflections for refinement. *R*-factors based on *F*^2^ are statistically about twice as large as those based on *F*, and *R*- factors based on ALL data will be even larger.

### Fractional atomic coordinates and isotropic or equivalent isotropic displacement parameters (Å^2^)

**Table:**

	*x*	*y*	*z*	*U*_iso_*/*U*_eq_	Occ. (<1)
C1	0.60839 (11)	0.20945 (11)	0.0000	0.0465 (6)	
C2	0.67561 (12)	0.19085 (12)	0.0000	0.0513 (6)	
H2	0.6869	0.1456	0.0000	0.062*	
C3	0.72584 (12)	0.23863 (13)	0.0000	0.0532 (6)	
C4	0.70778 (13)	0.30566 (14)	0.0000	0.0579 (7)	
H4	0.7411	0.3383	0.0000	0.070*	
C5	0.64153 (13)	0.32504 (12)	0.0000	0.0562 (7)	
C6	0.59123 (12)	0.27666 (12)	0.0000	0.0522 (6)	
H6	0.5464	0.2894	0.0000	0.063*	
C7	0.49362 (12)	0.15957 (11)	0.0000	0.0506 (6)	
C8	0.45900 (11)	0.09187 (12)	0.0000	0.0543 (7)	
H8A	0.4728	0.0668	−0.1115	0.065*	0.50
H8B	0.4728	0.0668	0.1115	0.065*	0.50
C9	0.38416 (12)	0.09873 (11)	0.0000	0.0515 (6)	
H9A	0.3708	0.1242	0.1113	0.062*	0.50
H9B	0.3708	0.1242	−0.1113	0.062*	0.50
C10	0.34741 (11)	0.03335 (12)	0.0000	0.0491 (6)	
C11	0.23912 (14)	−0.01607 (15)	0.0000	0.0666 (8)	
H11	0.2470 (10)	−0.0432 (11)	0.118 (3)	0.080*	
C12	0.16867 (16)	0.0096 (2)	0.0000	0.0849 (10)	
H12A	0.139 (2)	−0.029 (2)	0.0000	0.102*	
H12B	0.1605 (12)	0.0349 (13)	0.126 (4)	0.102*	
C13	0.79850 (13)	0.21804 (16)	0.0000	0.0652 (7)	
H13A	0.8077	0.1916	−0.1110	0.078*	0.50
H13B	0.8078	0.1920	0.1118	0.078*	0.50
H13C	0.8263	0.2573	−0.0007	0.078*	
C14	0.62237 (18)	0.39835 (14)	0.0000	0.0829 (10)	
H14A	0.6403	0.4196	−0.1113	0.099*	0.50
H14B	0.6402	0.4196	0.1115	0.099*	0.50
H14C	0.5744	0.4024	−0.0003	0.099*	
N1	0.56095 (10)	0.15635 (10)	0.0000	0.0519 (6)	
H1N	0.5776 (15)	0.1170 (16)	0.0000	0.062*	
O1	0.46202 (9)	0.21173 (9)	0.0000	0.0821 (8)	
O2	0.37347 (9)	−0.02118 (8)	0.0000	0.0701 (7)	
O3	0.28217 (8)	0.04272 (8)	0.0000	0.0614 (6)	

### Atomic displacement parameters (Å^2^)

**Table:**

	*U*^11^	*U*^22^	*U*^33^	*U*^12^	*U*^13^	*U*^23^
C1	0.0444 (12)	0.0435 (12)	0.0516 (12)	−0.0050 (9)	0.000	0.000
C2	0.0475 (13)	0.0481 (13)	0.0583 (13)	−0.0009 (9)	0.000	0.000
C3	0.0465 (12)	0.0632 (15)	0.0498 (12)	−0.0103 (10)	0.000	0.000
C4	0.0567 (15)	0.0593 (15)	0.0578 (14)	−0.0177 (11)	0.000	0.000
C5	0.0621 (15)	0.0464 (13)	0.0601 (14)	−0.0105 (10)	0.000	0.000
C6	0.0494 (13)	0.0426 (12)	0.0645 (14)	−0.0036 (9)	0.000	0.000
C7	0.0433 (12)	0.0411 (12)	0.0673 (15)	0.0014 (9)	0.000	0.000
C8	0.0432 (13)	0.0422 (13)	0.0775 (16)	−0.0015 (9)	0.000	0.000
C9	0.0447 (13)	0.0425 (12)	0.0673 (15)	0.0006 (9)	0.000	0.000
C10	0.0430 (12)	0.0449 (12)	0.0594 (14)	0.0013 (9)	0.000	0.000
C11	0.0497 (14)	0.0559 (15)	0.094 (2)	−0.0124 (11)	0.000	0.000
C12	0.0490 (16)	0.094 (3)	0.112 (3)	−0.0109 (15)	0.000	0.000
C13	0.0460 (14)	0.0815 (19)	0.0681 (16)	−0.0094 (12)	0.000	0.000
C14	0.083 (2)	0.0443 (15)	0.121 (3)	−0.0118 (13)	0.000	0.000
N1	0.0424 (11)	0.0367 (10)	0.0768 (14)	−0.0013 (8)	0.000	0.000
O1	0.0481 (10)	0.0416 (10)	0.157 (2)	0.0021 (7)	0.000	0.000
O2	0.0514 (10)	0.0397 (10)	0.1193 (18)	0.0015 (7)	0.000	0.000
O3	0.0420 (9)	0.0458 (9)	0.0965 (14)	−0.0017 (7)	0.000	0.000

### Geometric parameters (Å, °)

**Table:**

C1—C6	1.383 (3)	C9—H9A	0.9700
C1—C2	1.391 (3)	C9—H9B	0.9700
C1—N1	1.420 (3)	C10—O2	1.205 (3)
C2—C3	1.382 (3)	C10—O3	1.314 (3)
C2—H2	0.9300	C11—H11^i^	1.00 (2)
C3—C4	1.384 (4)	C11—O3	1.453 (3)
C3—C13	1.506 (4)	C11—C12	1.495 (4)
C4—C5	1.376 (4)	C11—H11	1.00 (2)
C4—H4	0.9300	C12—H12B^i^	1.03 (3)
C5—C6	1.392 (3)	C12—H12A	0.98 (4)
C5—C14	1.511 (4)	C12—H12B	1.03 (3)
C6—H6	0.9300	C13—H13A	0.9600
C7—O1	1.216 (3)	C13—H13B	0.9600
C7—N1	1.344 (3)	C13—H13C	0.9600
C7—C8	1.516 (3)	C14—H14A	0.9600
C8—C9	1.498 (3)	C14—H14B	0.9600
C8—H8A	0.9700	C14—H14C	0.9600
C8—H8B	0.9700	N1—H1N	0.85 (3)
C9—C10	1.495 (3)		
			
C6—C1—C2	119.8 (2)	H9A—C9—H9B	107.6
C6—C1—N1	123.9 (2)	O2—C10—O3	123.7 (2)
C2—C1—N1	116.3 (2)	O2—C10—C9	125.1 (2)
C3—C2—C1	121.0 (2)	O3—C10—C9	111.17 (19)
C3—C2—H2	119.5	H11^i^—C11—O3	110.1 (12)
C1—C2—H2	119.5	H11^i^—C11—C12	109.4 (12)
C2—C3—C4	118.5 (2)	O3—C11—C12	106.2 (2)
C2—C3—C13	120.6 (3)	H11^i^—C11—H11	112 (3)
C4—C3—C13	120.9 (2)	O3—C11—H11	110.1 (12)
C5—C4—C3	121.4 (2)	C12—C11—H11	109.4 (12)
C5—C4—H4	119.3	H12B^i^—C12—C11	108.4 (14)
C3—C4—H4	119.3	H12B^i^—C12—H12A	106.9 (16)
C4—C5—C6	119.8 (2)	C11—C12—H12A	107 (2)
C4—C5—C14	121.0 (2)	H12B^i^—C12—H12B	118 (3)
C6—C5—C14	119.2 (3)	C11—C12—H12B	108.4 (14)
C1—C6—C5	119.6 (2)	H12A—C12—H12B	106.9 (16)
C1—C6—H6	120.2	C3—C13—H13A	109.5
C5—C6—H6	120.2	C3—C13—H13B	109.5
O1—C7—N1	123.9 (2)	H13A—C13—H13B	109.5
O1—C7—C8	121.7 (2)	C3—C13—H13C	109.5
N1—C7—C8	114.3 (2)	H13A—C13—H13C	109.5
C9—C8—C7	111.8 (2)	H13B—C13—H13C	109.5
C9—C8—H8A	109.2	C5—C14—H14A	109.5
C7—C8—H8A	109.2	C5—C14—H14B	109.5
C9—C8—H8B	109.2	H14A—C14—H14B	109.5
C7—C8—H8B	109.2	C5—C14—H14C	109.5
H8A—C8—H8B	107.9	H14A—C14—H14C	109.5
C10—C9—C8	114.1 (2)	H14B—C14—H14C	109.5
C10—C9—H9A	108.7	C7—N1—C1	129.0 (2)
C8—C9—H9A	108.7	C7—N1—H1N	116 (2)
C10—C9—H9B	108.7	C1—N1—H1N	115 (2)
C8—C9—H9B	108.7	C10—O3—C11	118.0 (2)
			
C6—C1—C2—C3	0.0	C7—C8—C9—C10	180.0
N1—C1—C2—C3	180.0	C8—C9—C10—O2	0.0
C1—C2—C3—C4	0.0	C8—C9—C10—O3	180.0
C1—C2—C3—C13	180.0	H11^i^—C11—C12—H12B^i^	−54 (2)
C2—C3—C4—C5	0.0	O3—C11—C12—H12B^i^	64.8 (16)
C13—C3—C4—C5	180.0	O1—C7—N1—C1	0.0
C3—C4—C5—C6	0.0	C8—C7—N1—C1	180.0
C3—C4—C5—C14	180.0	C6—C1—N1—C7	0.0
C2—C1—C6—C5	0.0	C2—C1—N1—C7	180.0
N1—C1—C6—C5	180.0	O2—C10—O3—C11	0.0
C4—C5—C6—C1	0.0	C9—C10—O3—C11	180.0
C14—C5—C6—C1	180.0	H11^i^—C11—O3—C10	−61.7 (13)
O1—C7—C8—C9	0.0	C12—C11—O3—C10	180.0
N1—C7—C8—C9	180.0		

Symmetry codes: (i) *x*, *y*, −*z*.

### Hydrogen-bond geometry (Å, °)

**Table:**

*D*—H···*A*	*D*—H	H···*A*	*D*···*A*	*D*—H···*A*
N1—H1N···O2^ii^	0.85 (3)	2.15 (3)	2.995 (3)	176 (3)

Symmetry codes: (ii) −*x*+1, −*y*, −*z*.
